# Supplementary material for: LungVis 1.0: an automatic AI-powered 3D imaging ecosystem unveils spatial profiling of nanoparticle delivery and acinar migration of lung macrophages
Source: Nat Commun. 2024 Nov 27;15:10138. doi: 10.1038/s41467-024-54267-1 (PMC11603200; doi:10.1038/s41467-024-54267-1)
Supplement: Supplementary file 1 — Supplementary Information [file 41467_2024_54267_MOESM1_ESM.pdf]

Supplementary Information

LungVis 1.0: an automatic AI-powered 3D imaging ecosystem unveils spatial profiling of nanoparticle delivery and acinar migration of lung macrophages

Lin Yang[1]*, Qiongliang Liu[1, 11], Pramod Kumar[1], Arunima Sengupta[1], Ali Farnoud[1], Ruolin Shen[2], Darya Trofimova[3, 4], Sebastian Ziegler[3, 4], Neda Davoudi[5, 6], Ali Doryab[1], Ali Önder Yildirim[1], Markus E. Diefenbacher[1, 7, 8], Herbert B. Schiller[1, 9], Daniel Razansky[5, 6], Marie Piraud[2], Gerald Burgstaller[1], Wolfgang G. Kreyling[1, 10], Fabian Isensee[3, 4], Markus Rehberg[1], Tobias Stoeger[1], Otmar Schmid[1]*

[1]Institute of Lung Health and Immunity (LHI), Helmholtz Munich, Comprehensive Pneumology Center (CPC-M), Member of the German Center for Lung Research (DZL), Munich, Germany

[2]Helmholtz AI, Helmholtz Munich, Munich, Germany

[3]Helmholtz Imaging, German Cancer Research Center (DKFZ), Heidelberg, Germany

[4]Division of Medical Image Computing, German Cancer Research Center (DKFZ), Heidelberg, Germany

[5]Institute of Pharmacology and Toxicology and Institute for Biomedical Engineering, Faculty of Medicine, University of Zurich, Zurich, Switzerland

[6]Institute for Biomedical Engineering, Department of Information Technology and Electrical Engineering, ETH Zurich, Zurich, Switzerland

[7]Ludwig Maximilian University Munich, Munich, Germany

[8]DKTK Munich, Munich, Germany

[9]Research Unit for Precision Regenerative Medicine (PRM), Helmholtz Munich, Munich, Germany

[10]Institute of Epidemiology (EPI), Helmholtz Munich, Munich, Germany

[11] Present address: Department of Thoracic Surgery, Shanghai General Hospital, Shanghai Jiao Tong University School of Medicine, Shanghai, China.

Correspondence and requests for materials should be addressed to L.Y. (lin.yang@helmholtz-munich.de) or to O.S. (otmar.schmid@helmholtz-munich.de).

Supplementary Methods

Fluorescence stability measurement of melamine resin particles in cell culture

The murine alveolar macrophages cell line MH-S cells were purchased from ATCC. MH-S cells were cultured in RPMI-1640 cell culture medium supplemented with 10% FBS (Gibco, Germany) and 1% Penicillin-Streptomycin solution (Gibco, Germany) at 37 °C with 5% CO_2. Cells were seeded in 24-well plates at the density of 0.1×10^6 cells/well until reaching ca.60-70% confluency. MF Particles were added to the 500 µL cell culture medium with a concentration of 10 µg/mL in each well and cultured over a period of four days. For the controls, MF particles were also incubated with pure cell culture medium and distilled water under the same condition. Samples were collected and centrifuged at 10,000 rpm for 10 mins to separate the cell pellets and supernatant at time intervals of 0 h, 24 h, and 4 d post-exposure. The MF fluorescence intensities in cell pellets and supernatant were measured under the wavelength of 636/685 nm with a 10 nm bandwidth of optical filters in a standard plate reader (Tecan Safire 2), to detect the intact particles and possible free dyes released from particles, respectively. The ratio of MF total fluorescence intensity in cell culture normalized to that in water and the ratio of MF fluorescence intensity in cell pellets normalized to total intensity in cell pellets and supernatant were determined (Supplementary Fig. 4). Data represents the mean and standard deviations (SD) of two independent experiments.

Quantitative analysis of NP signals in adjacent acini

Inter-acinar exchange of NPs is expected to be most reliably demonstrated and quantified in those regions of the lung, where acini with extremely high NP dose are in close proximity to acini with extremely low NP dose. This is due to the fact that the clearance rate from high-dose acini is high and potential transport of those NPs into an adjacent low-dose acinus is most likely and most feasible detectable via imaging-based quantification.

To determine the NP fluorescence signal in acinar regions with both high and no/low NP enrichment, representative alveolar areas of identical size were selected from adjacent acini using ovals or rectangles in ITLI and VAAD lungs at both 24 h and 14 d post-exposure. Histogram profiling of these selected areas was performed in ImageJ, displaying the intensity of each pixel against the number of pixels after identical thresholding of tissue autofluorescence. A pie chart was used to indicate the proportion of particle signals from the high NP-enriched regions compared to the no/low NP-enriched regions.

Supplementary Figures

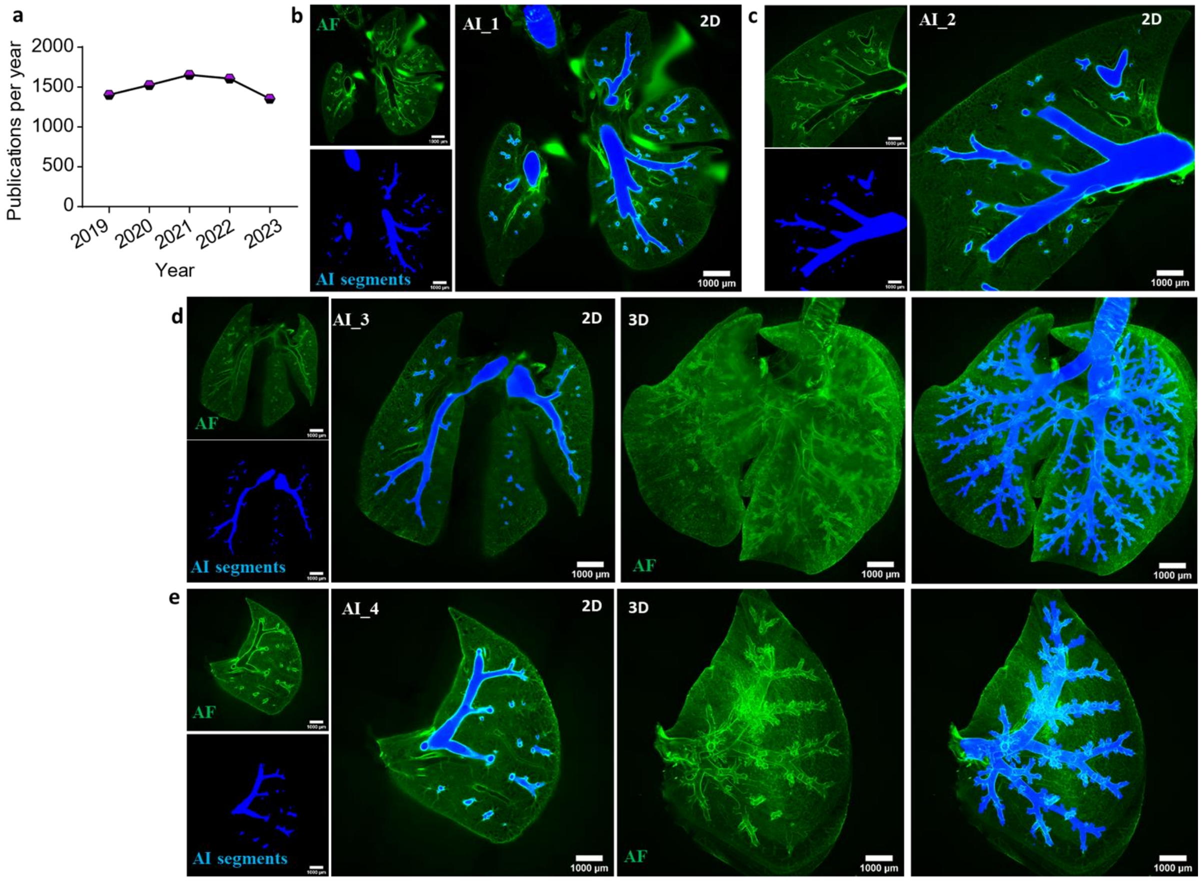

Supplementary Fig. 1 | a The number of publications per year associated with pulmonary substance delivery searched with topics of “inhalation exposure” or “pulmonary delivery” or “lung instillation” or “intratracheal instillation” or “nose only” or “intranasal delivery” or "oropharyngeal delivery" or “lung administration” or “lung exposure” or “aerosol delivery” or “lung injection” or “intranasal aspiration” or "oropharyngeal administration” or “intubated instillation” or “instilled delivery” or “inhaled delivery” or “inhaled aerosol” or "oropharyngeal aspiration" or "oropharyngeal exposure" or "inhalation" or "nasal delivery" or "nasal inhalation" or "oral inhalation" or "oral exposure", and “mice” but not “patients” in the Web of Science core collection that have been published as forms of research articles, reviews, letters during the last 5 years (2019-2023, mean 1508 papers per year). **b-f** Exemplary AI segmented results of complete lung airways and methodological illumination for determination of B/A deposition ratio. **b** and **c** show representative 2D views of a whole lung or a single lobe (of the lungs displayed Fig. **1d**) with imaging artifacts like imaging shadow, obscure airways, and bright blood vessels. **d** and **e** display the excellent lung airway segmentations from the lungs with imaging artifacts such as blurring effects and uneven light illumination, which can be overcome by the data-centric active-learning AI approach. Source data are provided as a Source Data file.

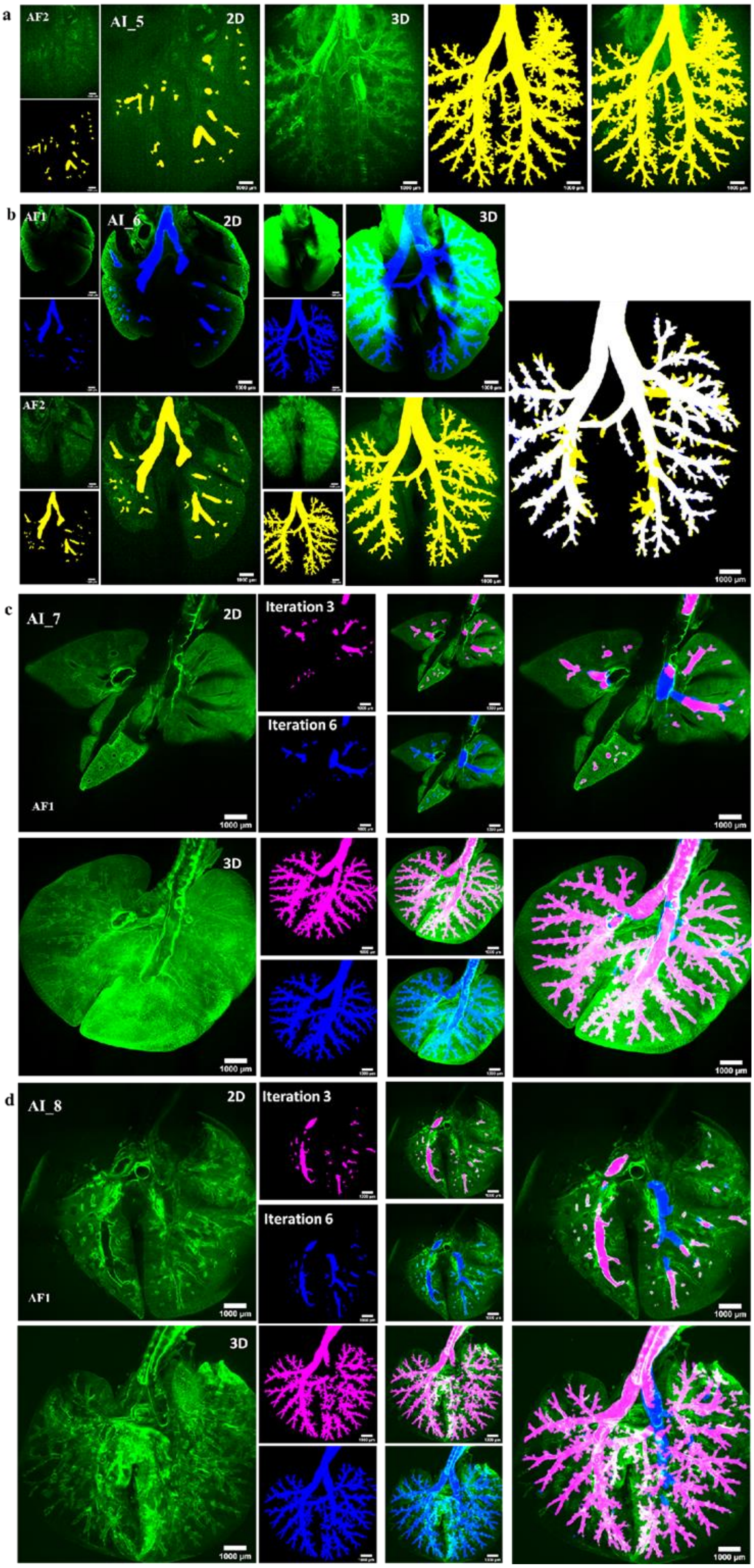
a
AF2
AI_5
2D
3D
b
AF1
AI_6
2D
3D
AF2
1000 µm
c
AI_7
2D
Iteration 3
Iteration 6
AF1
1000 µm
3D
1000 µm
d
AI_8
2D
Iteration 3
Iteration 6
AF1
1000 µm
3D
1000 µm

Supplementary Fig. 2 | Active learning AI-driven approach to whole-lung segmentation achieved high-quality labeling of lung airways from specimens with different kinds of imaging artifacts. a The lung specimen scanned in the near-infrared (AF2) channel showed variable signal-to-noise ratio, air-bubble cased false structures, very weak fluorescence intensity, and obscured airways. **b** Comparison of AI results generated from visible AF1 and AF2 channels in a lung sample with poor center illumination (dark regions) due to optically absorbing blood residuals, indicating that AF2 could gain better AI results due to deep light penetration in near-infrared wavelength. **c** and **d** Some lung specimens displayed very poor imaging qualities like inconsistent illumination, obscured airways, false structures, and air-bubbles caused gray structure. For those images the active learning AI approach with more corrected samples and with method improvement (such as Gaussian Noise, local contract transform, local brightness transform) provides better airway labeling (segmentation) quality (better AI results for iteration 6 than 3). Representative data from 78 lungs applied in this study.

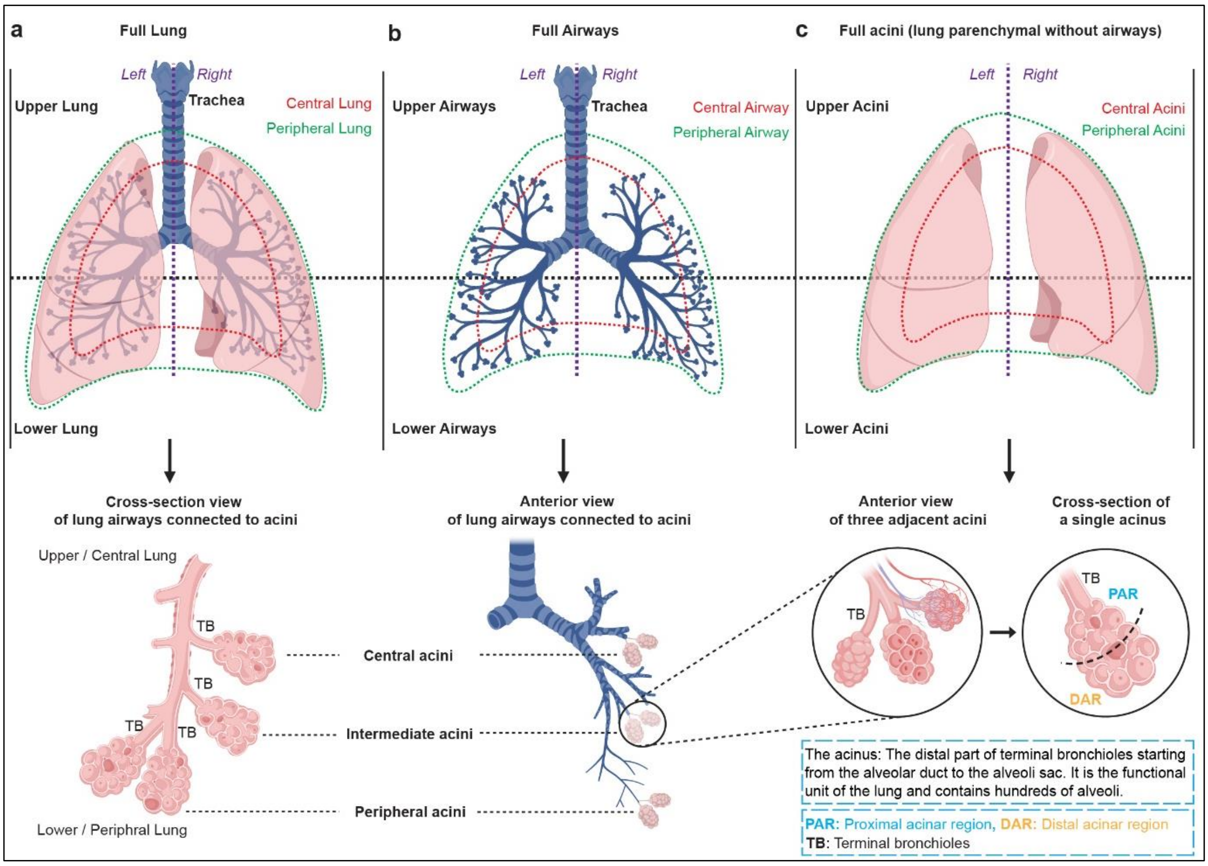

Supplementary Fig. 3 | The lung terminology used in this study. Top panels: The segregation of the upper and lower half of the lung (ca. 50:50 projected area), which include airways and acini, is indicated by the black dashed horizontal line. Analogously, the segregation between the left and right half of the lung is indicated by the purple dashed vertical line. The segregation of the central and peripheral half of the lung, where the central lung is directly connected to the trachea, is indicated by the red dashed line. Lower panels: we discriminate between central, intermediate, and peripheral acini along the bronchial tree. The terms (full) airways and entire bronchial tree are used interchangeably in this study. Each acinus is classified into about 50% proximal acinar region (PAR) and 50% distal acinar region (DAR). In general, the proximal part of any part of the lung is the one closer to the trachea. This unified terminology would facilitate a straightforward comprehension of NP deposition profiles in whole lung enabled with LungVis 1.0. Images created partially with Biorender.

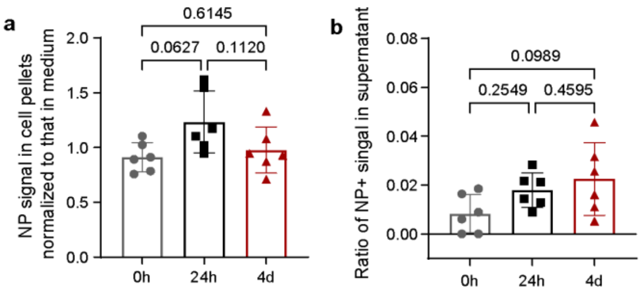

Supplementary Fig. 4 | Quantitative measurement of fluorescence stability of melamine resin (MF) particles in MH-S cells (murine macrophage cell line) for up to four days. MF particles at the concentration of 10 µg/mL were incubated with MH-S cells in 500 µl culture medium. At 0h, 24h and 4d the culture medium and cells were collected and centrifuged at 10,000 rpm to separate and detect the NP signals in cell pellets and supernatant. **a** MF NP fluorescence intensity in cell pellets normalized to the fluorescence intensities of the nominally expected NP concentration in cell culture medium at each time point. **b** Ratio of NP intensity in the supernatant to the total NP intensity from cell pellets and supernatant. Data are presented as mean values ± SD. Statistical comparisons were performed with one-way ANOVA followed by Holm-Sidak multiple comparisons tests. n = 2, independent biological replicates (3 technical replicate each). Source data are provided as a Source Data file.

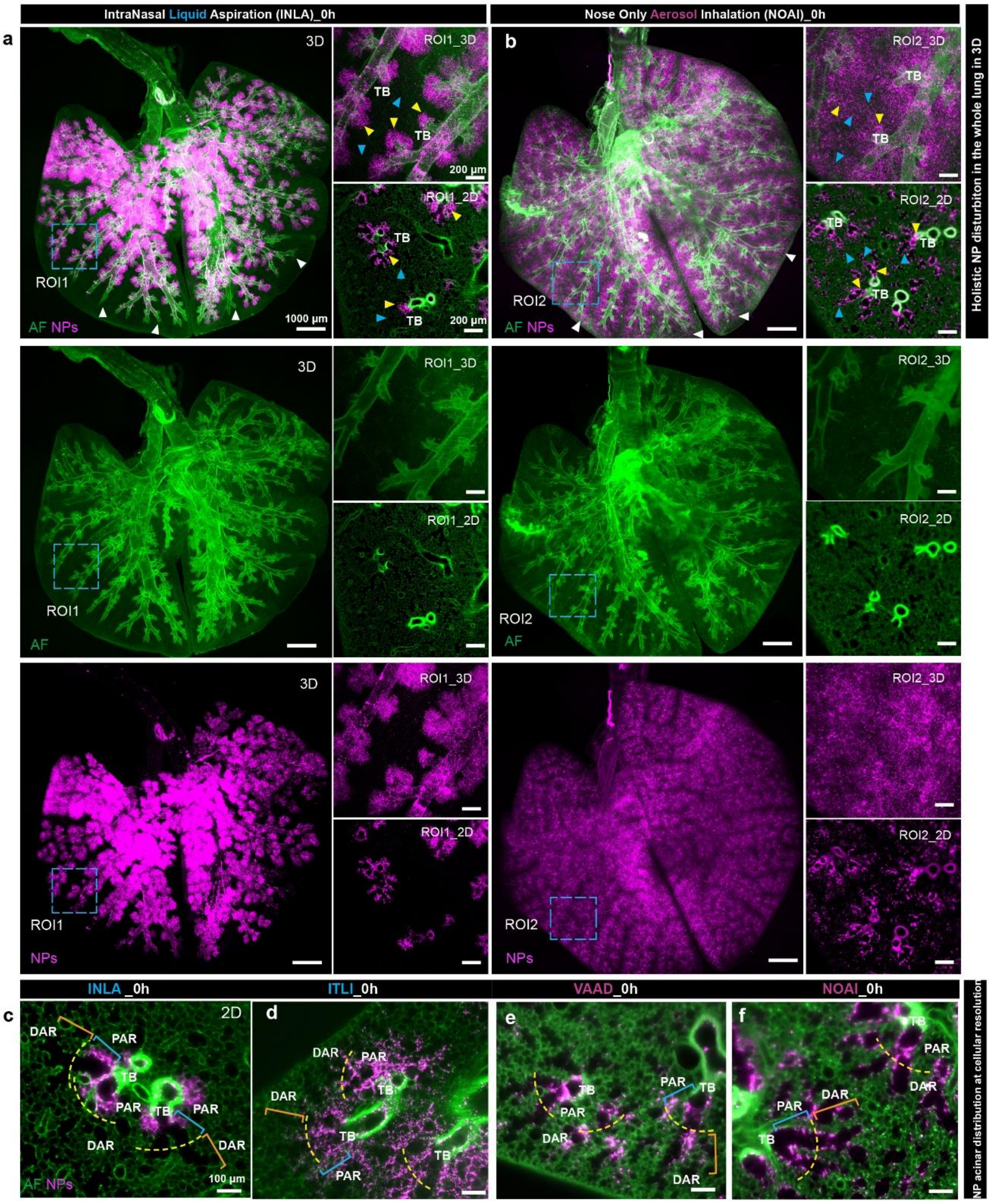

Supplementary Fig. 5 | a and **b** 3D merged and single channel view of lung morphology and NP distribution in lungs of mice immediately after intranasal liquid aspiration (INLA_0h) and nose-only aerosol inhalation (NOAI_0h), respectively. These observations were made from the LSFM raw data without AI processing. Arrow heads in white indicate the lower airways and peripheral acini. Arrow heads in yellow and blue indicate the proximal acinar regions (PAR) and distal acinar regions (DAR), respectively. **c-f** High-resolution 2D view of NP intra-acinar deposition patterns in the lungs after 4 types of delivery route. TB: terminal bronchioles. Within acini the proximal and distal acinar regions (PAR and DAR) are artificially separated by the yellow line. Representative data from independent biological replicates (INLA: n = 3, NOAI: n = 4).

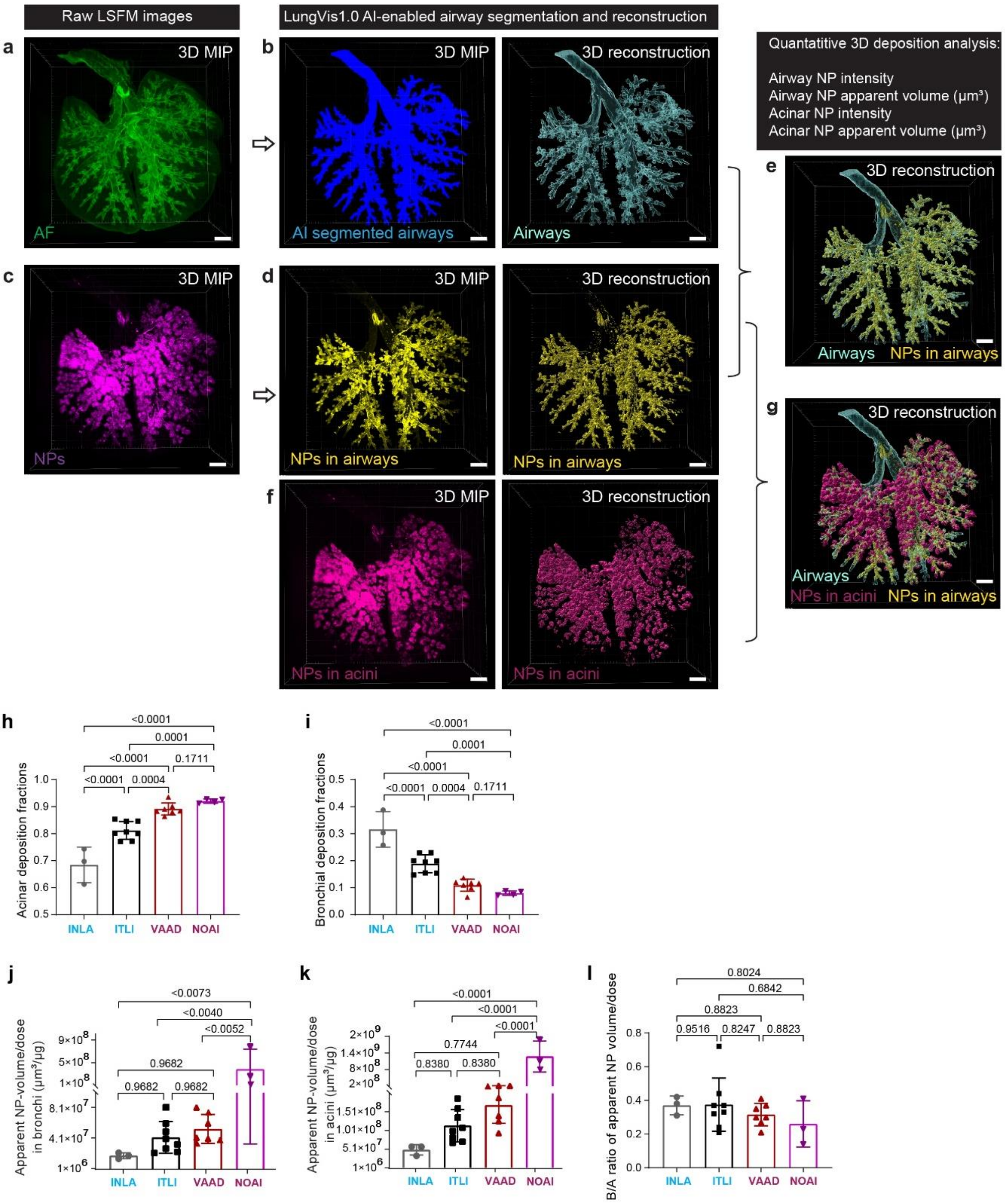

Supplementary Fig. 6 | Holistic assessment of spatial NP deposition profiles is achieved through airway segmentation and NP intensity-based quantitative analysis. **a-g** The methodology illustration for determining bronchial and acinar NP deposition fractions and NP agglomeration state. **h-l** The corresponding quantitative results. The raw LSFM autofluorescence image (**a**) was used to extract airway masks using LungVis 1.0 AI pipelines, enabling the reconstruction of the 3D airway structure (**b**). By overlaying the AI-driven airway masks with raw NP signals slice-by-slice over the entire lung LSFM stack (**c**), the delivered NPs in airways can be selectively visualized in 3D maximum intensity projection (MIP) and reconstructed images (**d**). Subsequently, the airway (bronchial) NP fluorescence intensity and apparent NP-positive volume can be determined in the 3D reconstructed NP-positive airway areas (**e**, in yellow) using Imaris or ImageJ. Similarly, NPs in acini (excluding airways) can be selectively visualized in 3D MIP and reconstructed 3D images (**f**), allowing for the determination of acinar NP fluorescence intensity and apparent NP-positive volume in 3D reconstructed NP-positive

acinar regions (**g**, in magenta: NPs in acini). **a - g** Scale bars: 1000 µm.
h and **i** The relative bronchial or acinar NP dose fractions were determined by normalizing the bronchial or acinar NP intensity to the total NP intensity, respectively. INLA: n = 3, ITLI: n = 8, VAAD: n = 7, NOAI: n = 4, biological replicates per group.
j and **k** The bronchial or acinar NP agglomeration state was calculated from the respective NP-positive volumes in 3D reconstructed images normalized to the NP dose in the bronchial or acinar region. This parameter is descriptive of NP spreading in the lung, i.e. low values indicate high NP agglomeration.
l The B/A ratio of NP agglomeration state. **j-l** INLA: n = 3, ITLI: n=8, VAAD: n = 7, NOAI: n = 3, biological replicates per group. **h-l** Data are presented as mean values ± SD, calculated using one-way ANOVA with Holm-Šídák's multiple comparison test, p values indicated in the graphs. Source data are provided as a Source Data file.

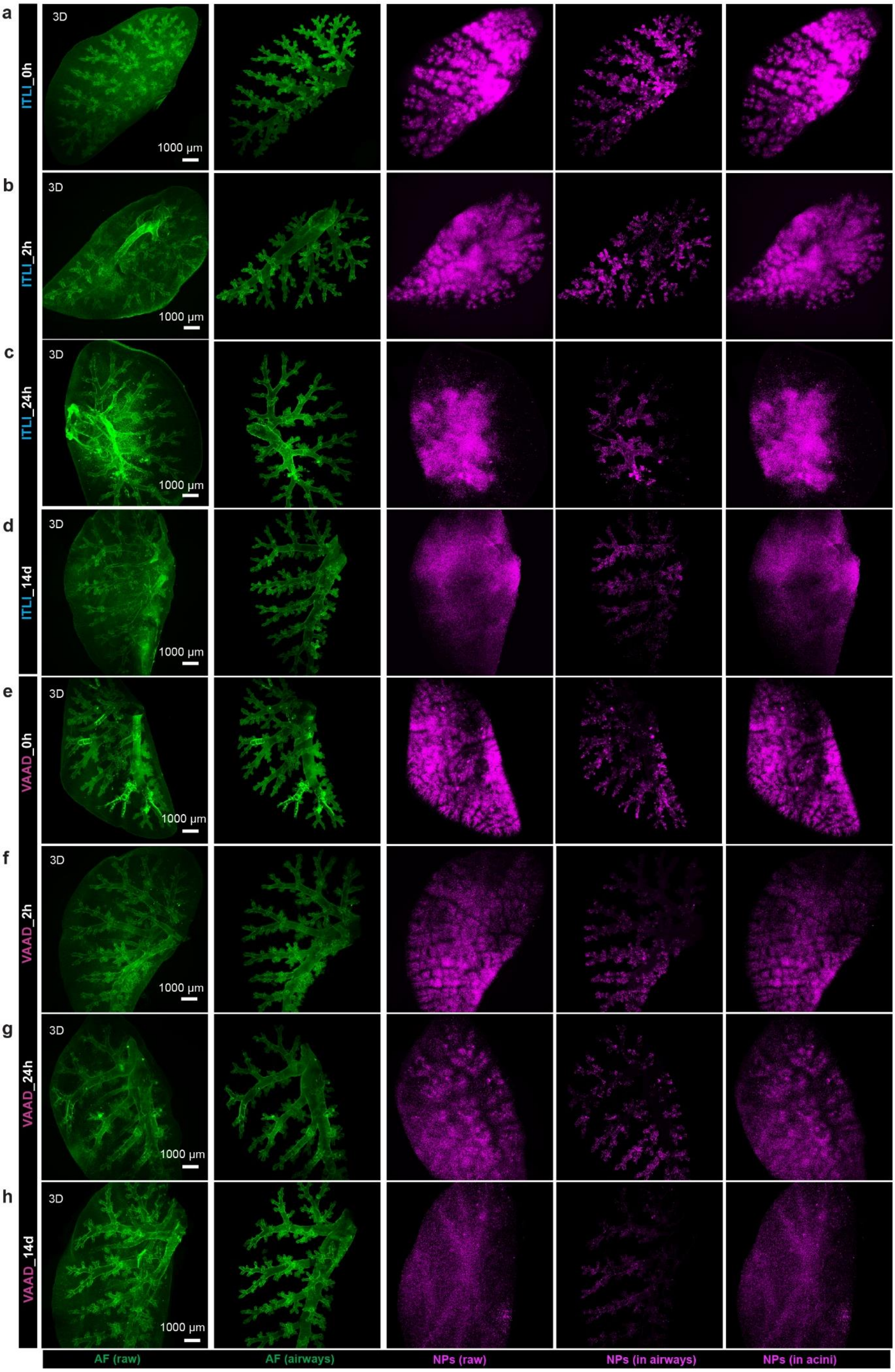
a
ITLI_0h
b
ITLI_2h
c
ITLI_24h
d
ITLI_14d
e
VAAD_0h
f
VAAD_2h
g
VAAD_24h
h
VAAD_14d
3D
1000 µm
AF (raw)
AF (airways)
NPs (raw)
NPs (in airways)
NPs (in acini)

Supplementary Fig. 7 | Single-channel 3D view of left lung lobes as raw signal and as LungVis 1.0-separated lung airways for both channels (tissues in green and NPs in magenta), depicting the time-resolved NP distribution in ITLI and VAAD lung airways or acini. Representative data from n=8 ITLI_0h, n=3 ITLI_2h, n=8 ITLI_24h, n=5 ITLI_14d, n=7 VAAD_0h, n=3 VAAD_2h, n=6 VAAD_24h, and n=5 VAAD_14d, independent biological replicates per group.

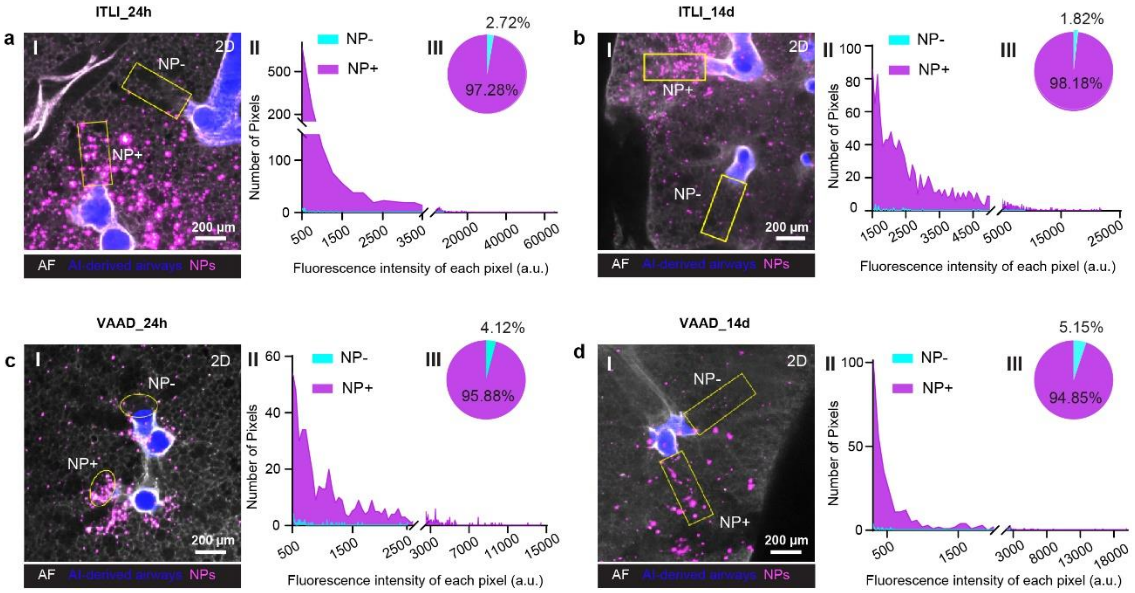

Supplementary Fig. 8 | Quantitative analysis of NP fluorescence signal in adjacent acini in the ITLI and VAAD lungs at 24h and 14d post-delivery. Representative acinar regions were selected to demonstrate the substantial difference of NP signals in adjacent acini (20-50-fold difference). Regions of interest were placed at the entrance of the acini along the centerline of the alveolar duct directly connected to the terminal bronchioles, where the NP dose is highest. The yellow ovals or rectangles (I) indicate the selected acinar regions where the NP fluorescence signals in adjacent high NP-dose regions (NP+) and no/low NP-dose regions (NP-) were quantified. The data is quantitatively presented using histogram profiles (II) and pie charts (III). a.u. indicate arbitrary unit. Data were quantified with n=2 biological replicates for each delivery method. Source data are provided as a Source Data file.

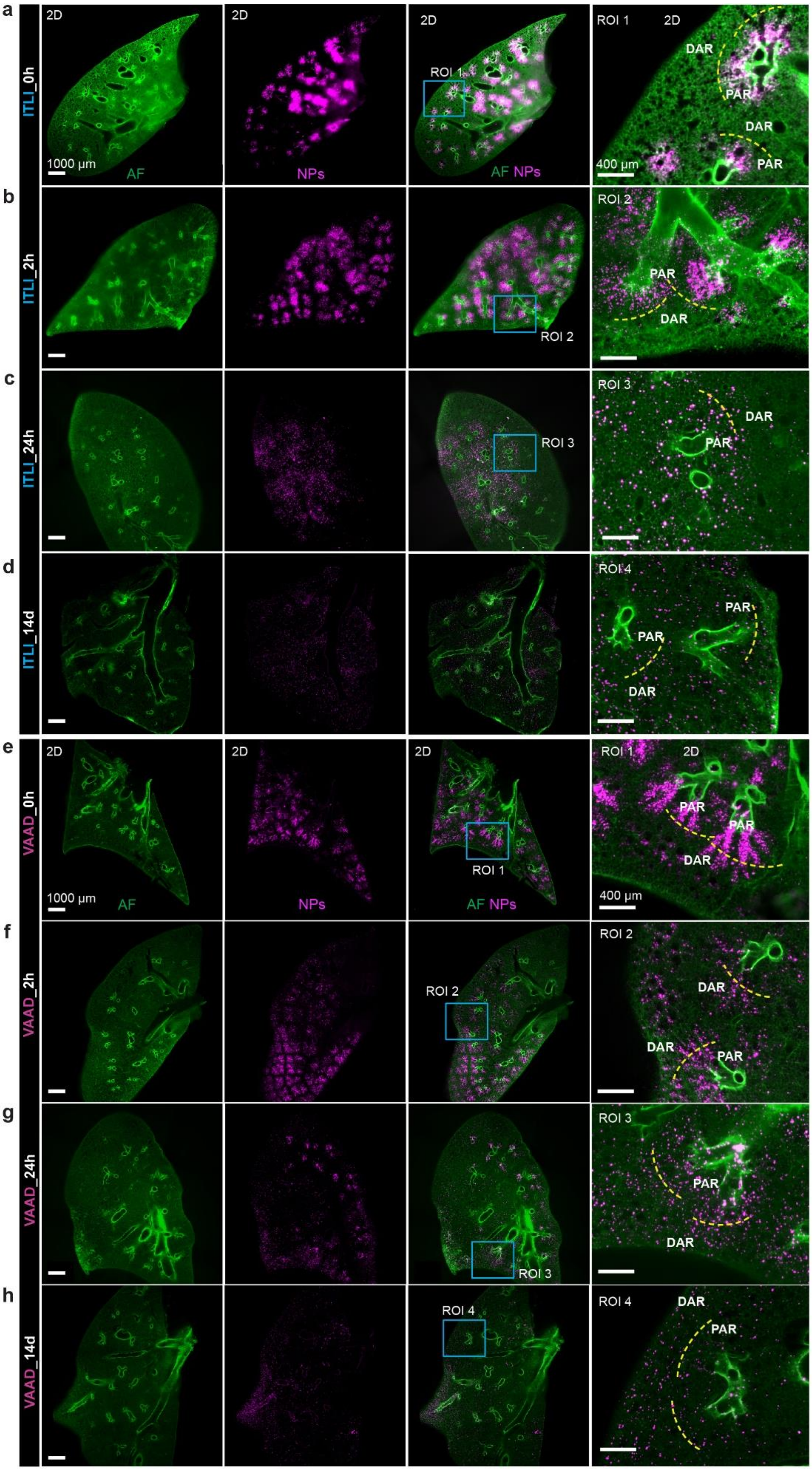

Supplementary Fig. 9 | Single-slice (single channel and merged) view of particle distribution pattern in mouse lungs over a time course ranging from 0h to 14d after intratracheal liquid instillation (ITLI, a-d) and ventilator-assisted aerosol delivery (VAAD, e-h). Representative data from n=8 ITLI_0h, n=3

ITLI_2h, n=8 ITLI_24h, n=5 ITLI_14d, n=7 VAAD_0h, n=3 VAAD_2h, n=6 VAAD_24h, n=5 VAAD_14d, independent biological replicates per group.

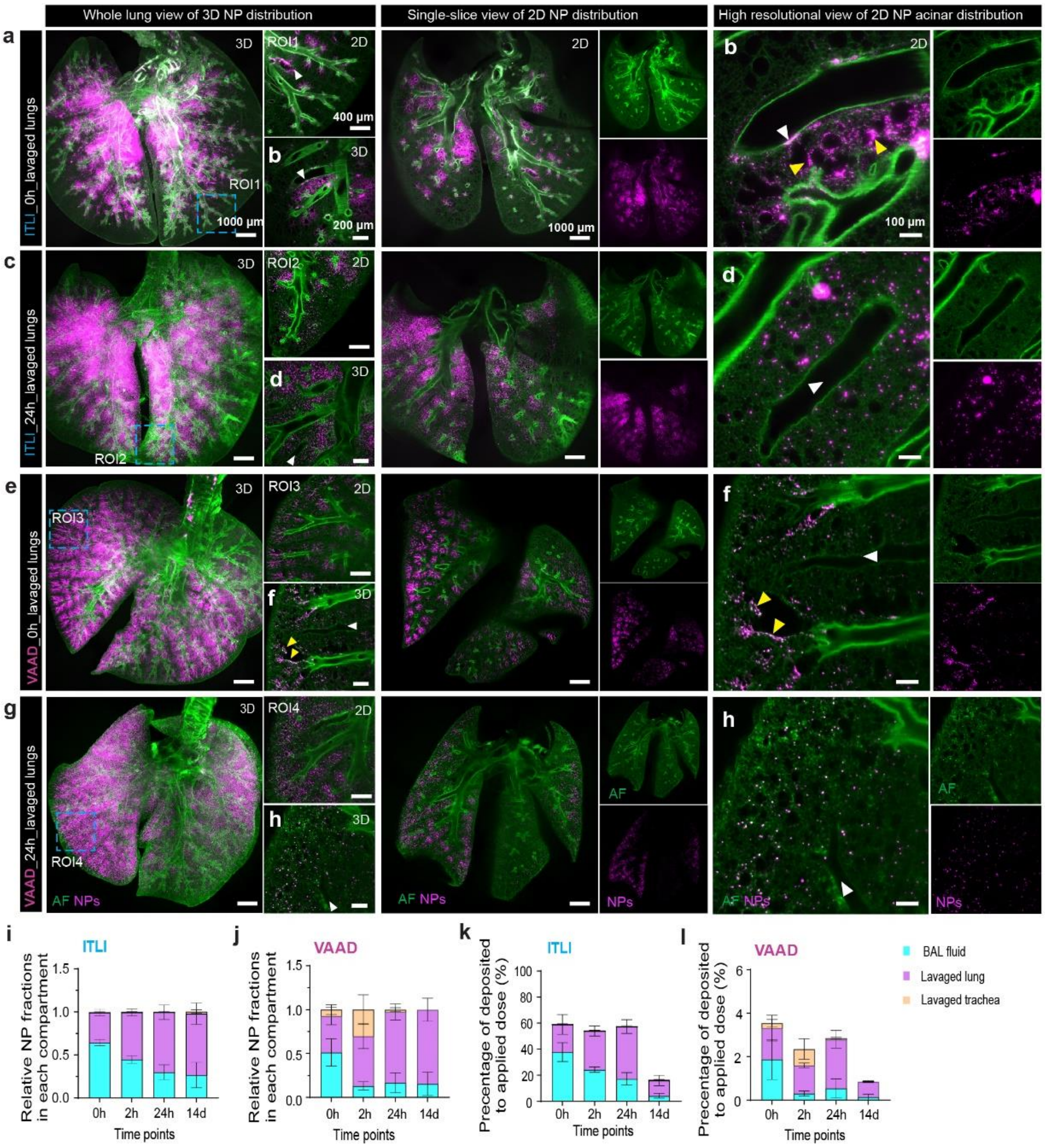

Supplementary Fig. 10 | Visualization of NP distribution and quantification of NP dose in lavaged lungs.

a-h Whole-lung and single-slice views of lavaged lungs post 0 h and 24 h ITLI and VAAD administrations. White arrowheads indicate blood vessels and NPs nearby, yellow arrowheads refer to regions of high NP agglomerations in the epithelium. The relative NP fraction (**i**, **j**) and the percentage of retained to applied doses in different lung compartments (BAL fluid, lavaged lungs, and lavaged trachea, **k**, **l**). n = 3, independent biological replicates per group. Data are presented as mean values ± SD, and Source data are provided as a Source Data file.

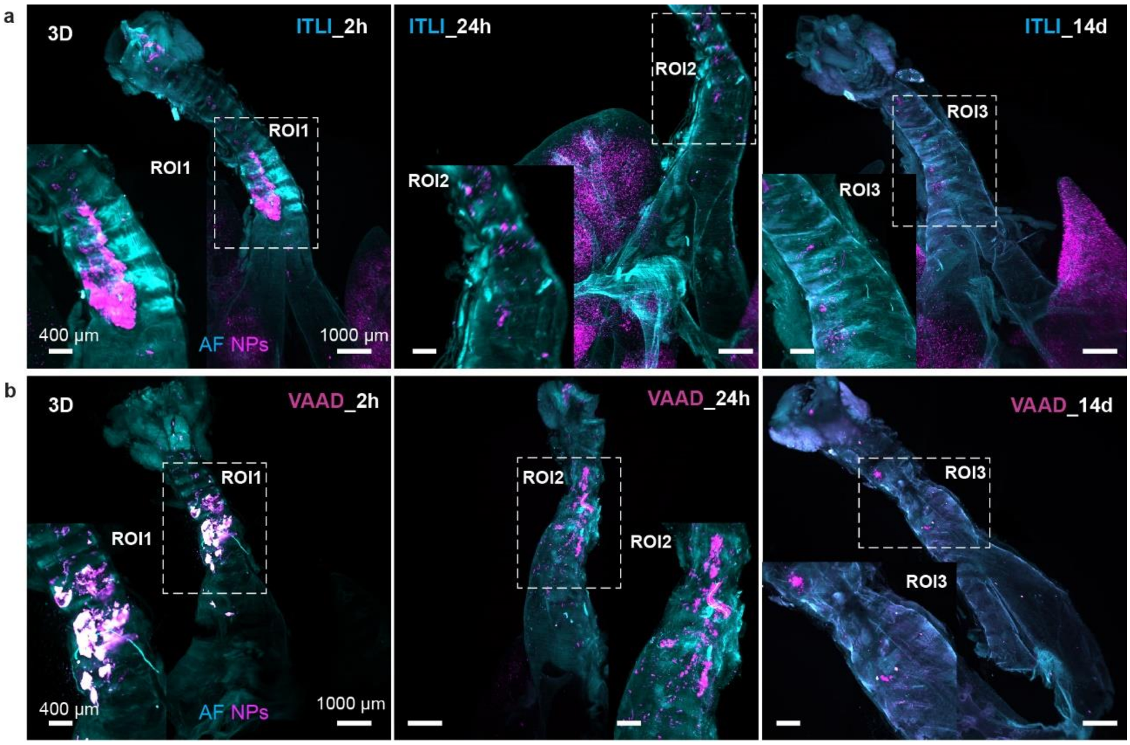

Supplementary Fig. 11 | longitudinal observation of particle deposition and clearance in mouse trachea post ITLI and VAAD applications at time points of 2 h, 24 h, and 14 d. Representative data from n = 3, independent biological replicates per group.

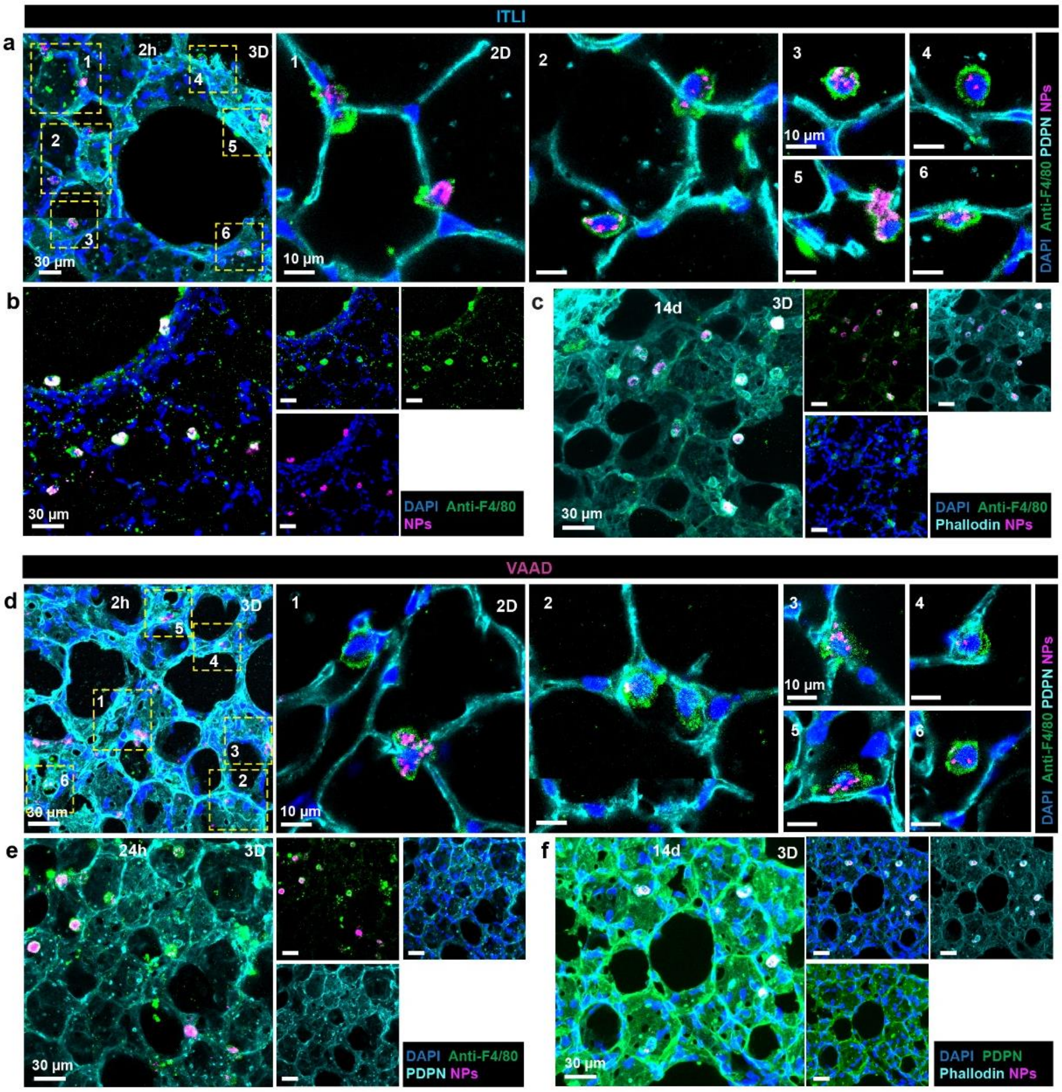

Supplementary Fig. 12 | Cellular resolution view of acinar localization and cellular uptake of NPs (mainly F4/80^{+} macrophages, TRMs) in PCLS using *in situ* immunofluorescence staining of mouse lungs after ITLI (a-c) or VAAD (d-f) application at time points of 2 h, 24 h, and 14 d. a and **d** show exemplary single-alveolus observations of NP-laden macrophages across multiple locations in 2h ITLI and VAAD lungs. Analogous images for 24 h and 14 d time points are presented in panels **b** and **e** (24 h) and panels **c** and **f** (14 d). Representative data from n = 5 ITLI_2h, n = 7 ITLI_24h, n = 5 ITLI_14d, n = 4 VAAD_2h, n = 7 VAAD_24h, n = 5 VAAD_14d, independent biological replicates per group.

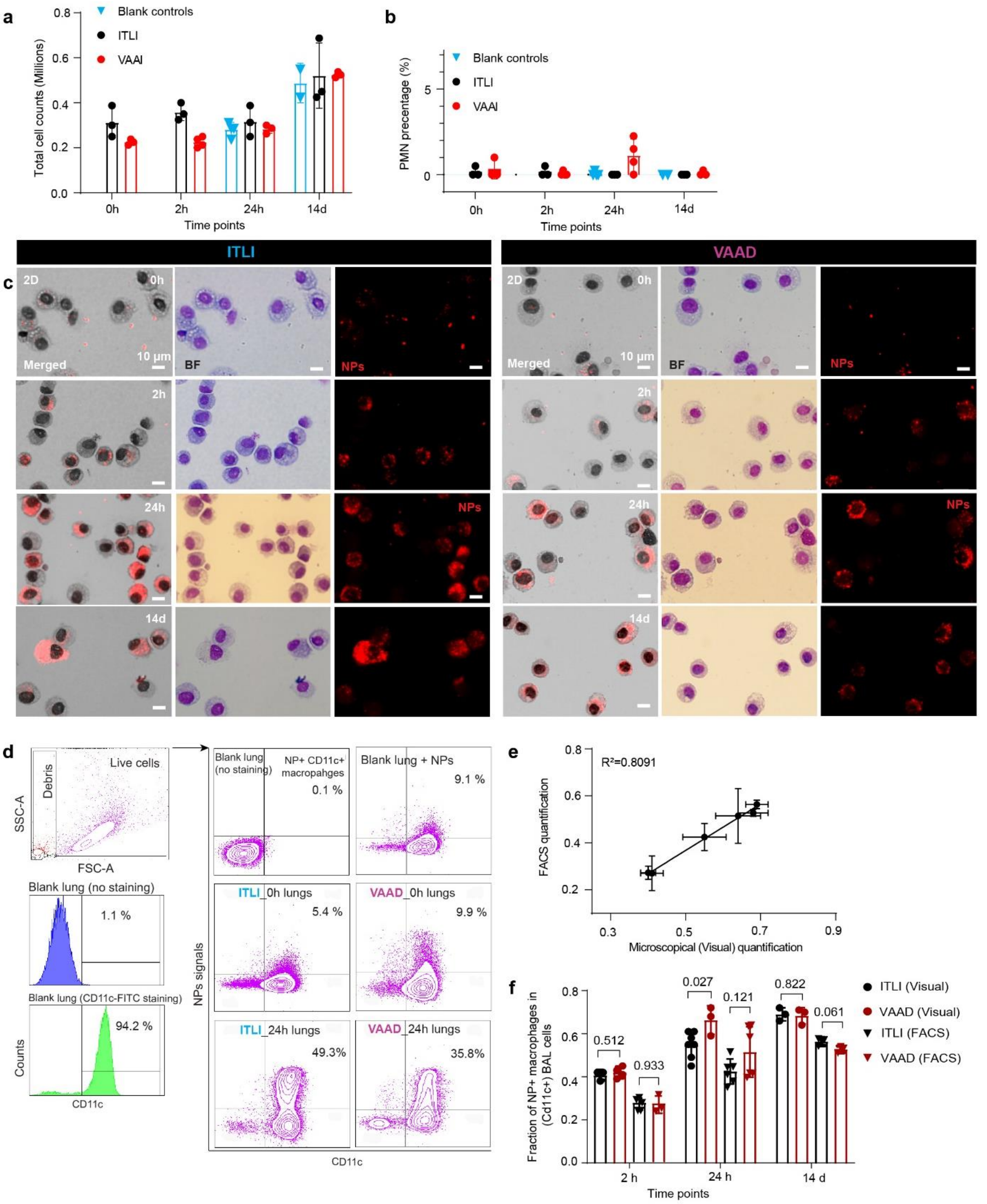

Supplementary Fig. 13 | Quantitative analysis of BAL cells obtained from 0h, 2h, 24h, and 14d ITLI and VAAD lungs. a, **b** Total cell count and PMN cell count at four time points post VAAD and ITLI deliveries. n= 4 Blank_24h, n=2 blank_14d, n=3 ITLI_0h, n=3 ITLI_2h, n=3 ITLI_24h, n=3 ITLI_14d, n=3 VAAD_0h, n=4 VAAD_2h, n=3 VAAD_24h, n=3 VAAD_14d, independent biological replicates per group. **c** Representative BAL cell images comprise both bright-field images (Grünwald-Giemsa stained) and fluorescence of NPs at 0h, 2h, 24h, and 14d post VAAD and ITLI deliveries. **d** Flow cytometry gating strategy for NP$^+$ CD11c$^+$ BAL cells obtained from 24h post VAAD and ITLI deliveries. **e** Linear correlation of two quantitative methods (visual examination and FACS quantification) for detection of NP$^+$ macrophages in BAL fluid (r^2= 0.8091). **f** The fractions of NP$^+$ macrophages to CD11c$^+$ macrophages (fluorescence activated cell sorting (FACS) analysis) or total macrophages (visual examination: microscopy of May-Grünwald-Giemsa stained cells) in BAL cells at three time points. **d**, **f** n = 5 ITLI_2h, n = 6 ITLI_24h, n = 3 ITLI_14d, n = 3 VAAD_2h, n = 6 VAAD_24h, n = 3 VAAD 14d, independent biological replicates (for

FACS experiments). Data are presented as mean values ± SD. Statistical comparisons were performed using unpaired two-tailed t-test (**f**). Source data are provided as a Source Data file.

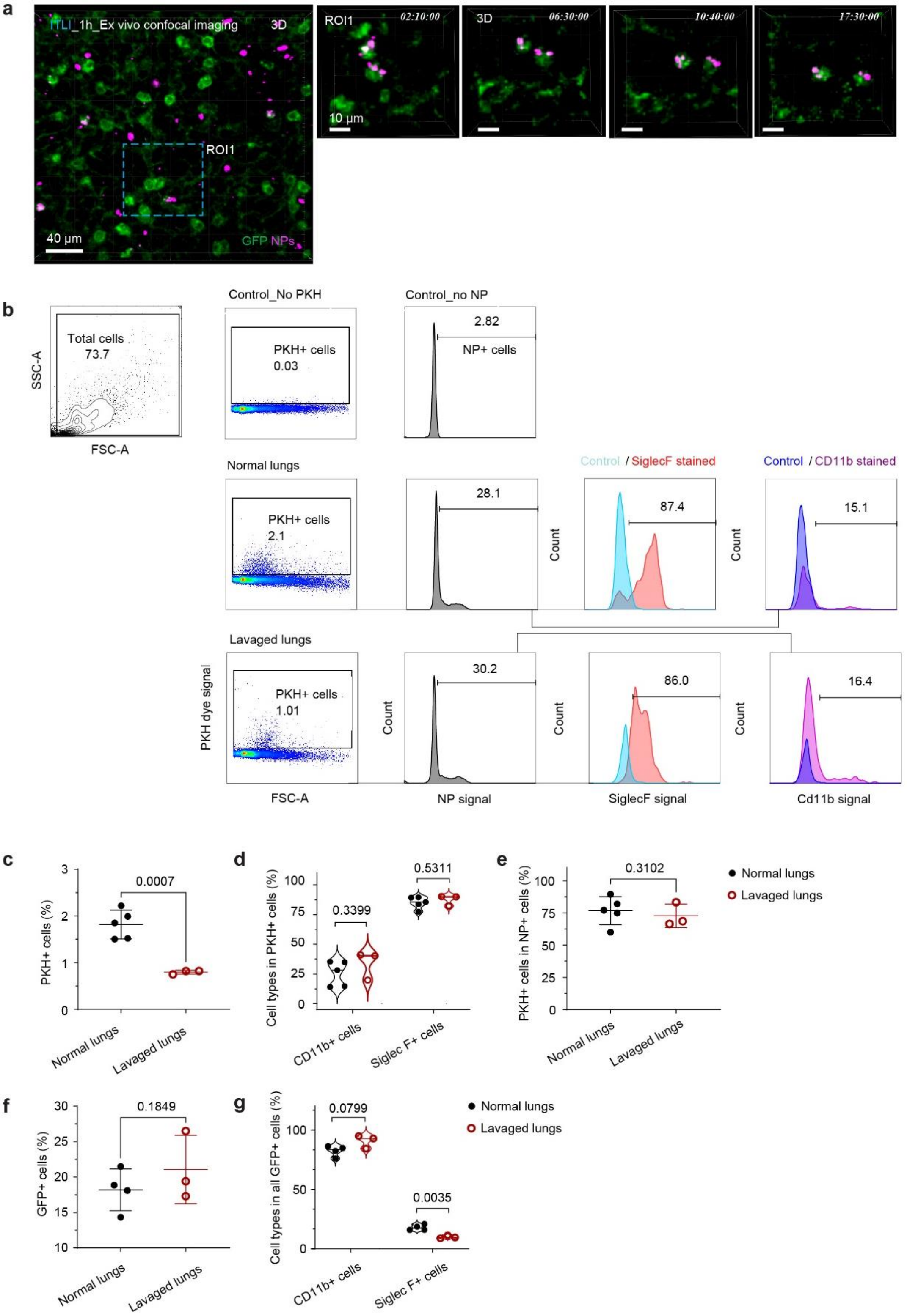

Supplementary Fig. 14 | a *Ex vivo* lung living microscopy depicted the migration of GFP-expressing, NP-laden macrophages in the PCLS punches within 18 h after ITLI administrations using laser scanning confocal microscopy. n = 4, independent biological replicates.

b Exemplary cell population analysis of $PKH^{+}NP^{+}$ macrophages in normal and lavaged WT mice and

quantifications displayed in **c-e**.

c Fractions of PKH$^+$ cells to total cells in normal and lavaged lungs. **d** Fractions of CD11b$^+$ or SiglecF$^+$ in PKH$^+$NP$^+$ cells in normal and lavaged lungs. **e** The fractions of PKH$^+$ cells to all NP$^+$ cells in normal and lavaged lungs. n = 3 (lavaged) or 5 (normal), independent biological replicates.

f The fractions of GFP$^+$ cells to total cells in normal and lavaged mac-green mouse lungs. **g** The fractions of CD11b$^+$ or SiglecF$^+$ in GFP$^+$NP$^+$ cells in normal and lavaged lungs. n = 3 (lavaged) or 4 (normal), independent biological replicates. Data are presented as mean values ± SD, calculated using one-tailed unpaired t test (**c**, **e, f**) and multiple two-tailed unpaired t test with Holm-Šídák correction for multiple comparisons (alpha = 0.05) (**d, g**). Source data are provided as a Source Data file.

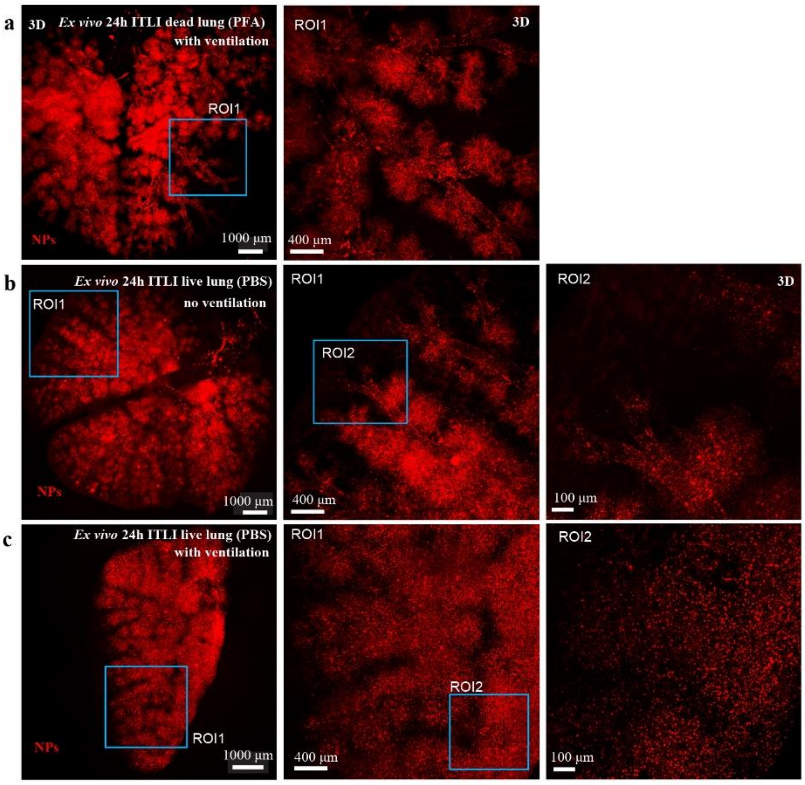

Supplementary Fig. 15 | Representative single-channel 3D view of NP whole-lung distribution/deposition pattern in *ex vivo* ITLI lungs post PFA fixation or PBS incubation and *ex vivo* ventilation for 24 h. Immediately after ITLI application of NPs into the mouse the lung was isolated from the animal, subjected to 3-4h of either PFA fixation (a and b) or PBS incubation (c) followed by 24h *ex vivo* physiological ventilation (no ventilation for b). Representative data from n=4, independent biological replicates per group.

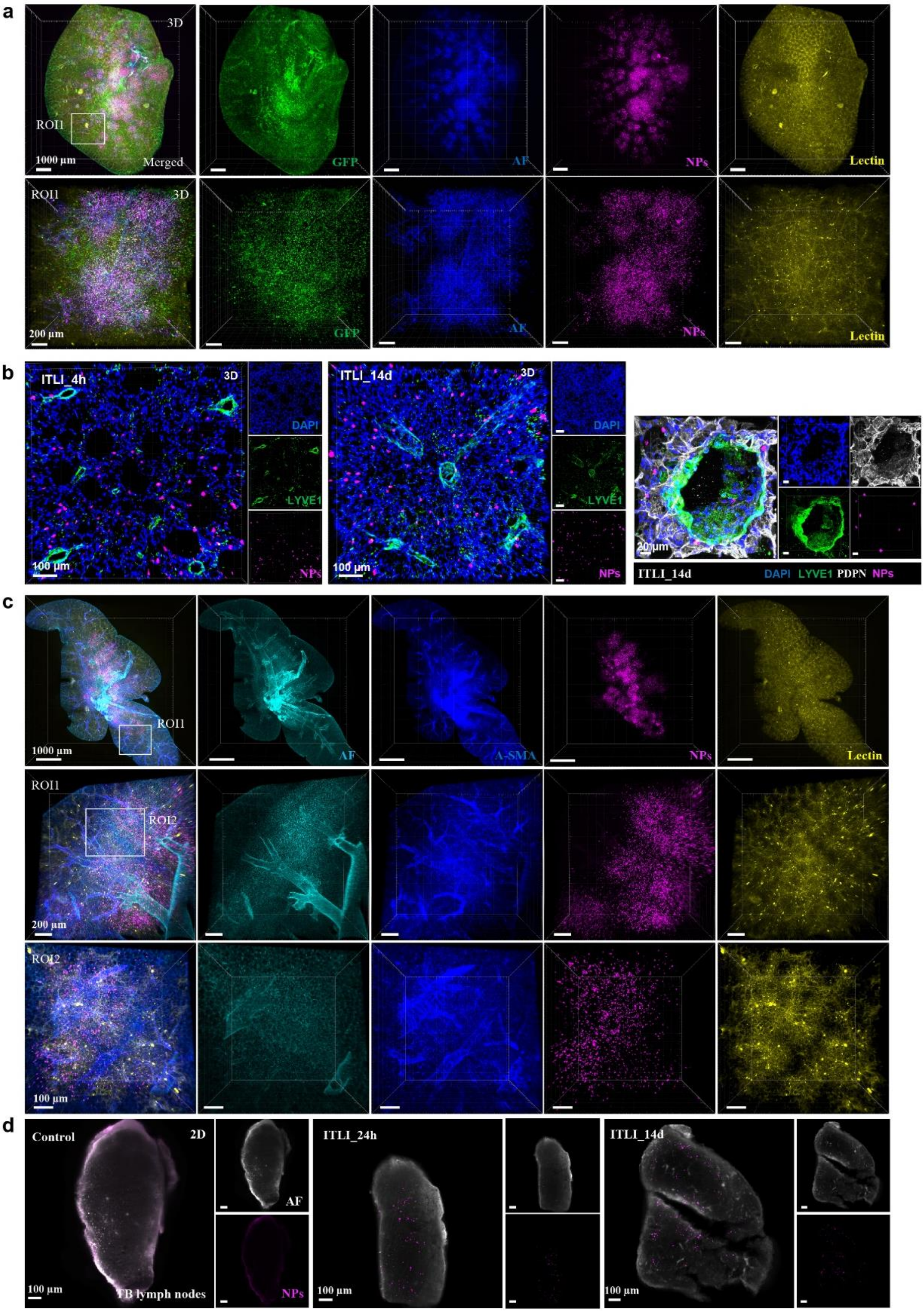

Supplementary Fig. 16 | Multispectral 3D imaging discloses NP distribution with respect to multiple lung networks. a Macroscale and microscale observations of NP distribution, macrophages, and lectin-labeled capillary network in an entire lung lobe of transgenic GFP-expressed mouse (CSF1R-GFP positive macrophages/monocytes). The same lung as shown in Fig. 6j.

b High-resolution visualization of PCLS imaged by confocal microscopy shows the proximity of NP localization with endothelial systems. n = 3, independent biological replicates.

c Multiple-scale observations of NP distribution, lectin-labeled vasculature, and alpha-SMA labeled network in an entire lung lobe of a wildtype mouse. n = 3, independent biological replicates.

d 2D sections of tracheobronchial lymph nodes obtained from blank mice or 24h and 14d ITLI exposed mice. n = 3, independent biological replicates.
