# Supplementary material for: LungVis 1.0: an automatic AI-powered 3D imaging ecosystem unveils spatial profiling of nanoparticle delivery and acinar migration of lung macrophages
Source: Nat Commun. 2024 Nov 27;15:10138. doi: 10.1038/s41467-024-54267-1 (PMC11603200; doi:10.1038/s41467-024-54267-1)
Supplement: Supplementary file 2 — Description of Additional Supplementary Files [file 41467_2024_54267_MOESM2_ESM.pdf]

Supplementary Data 1:

Descriptive and quantitative summary of pulmonary NP delivery and biokinetic features from 0 hour (immediate) to 14 days post-administration across four routes of pulmonary delivery.

Supplementary Movie 1: An exemplary holistic view of an INLA delivered lung with the manually annotation (ground truth) of lung airway.

Three-dimension (3D) co-mapping of lung morphology (green), INLA delivered NPs (magenta), manually annotated lung airway model (blue), separated airway (cyan), airway NPs (yellow), and acinar NPs (magenta) in a whole mouse lung enabled through LungVis 1.0. 3D reconstructions of separated airway, airway NPs, and acinar NPs are in transparent cyan, yellow, and magenta, respectively. It depicts the lung shown in Fig. 1b, Fig. 2e, Supplementary Fig. 5a, and Supplementary Fig. 6a-g.

Supplementary Movie 2: Exemplary active learning AI performance (LungVis 1.0) under a common visible light channel in a lung with poor imaging quality.

An exemplary case of data-centric active learning AI method gradually improving the segmentation performance of a full lung with out-of-center focus in the AF1 channel (ex/em=540/590, Fig. 1c). Lung morphology (autofluorescence, AF1) in green, iterations 1, 3, and 6 from AF1 channels are in cyan, purple, and yellow, respectively.

Supplementary Movie 3: Exemplary active learning AI performance (LungVis 1.0) in the near-infrared channel compared to a visible light channel in the lung with poor imaging quality.

An exemplary case of data-centric active learning AI methodology gradually improving the segmentation performance of a full lung with poor signal-to-noise ratio in the AF2 channel (ex/em=740/790 nm, Fig. 1c and Supplementary Fig. 2b). Lung morphology (autofluorescence AF2) in gray, iterations 1, 3, and 6 from AF2 channels are in cyan, purple, and yellow, respectively. Lung morphology in AF1 in green and corresponding iteration 6 in blue. VAAD delivered NPs in red.

Supplementary Movie 4: A perfect case of whole-lung airway segmentation resulted from LungVis 1.0.

This lung airway segmentation from the common visible light channel was obtained from our data-centric active learning AI model (Fig. 1d, whole lung). Lung morphology in green and iteration 6 in blue.

Supplementary Movie 5: An exemplary holistic view of a NOAI delivered lung.

3D co-mapping of lung morphology (green), NOAI delivered NPs (magenta), lung airway model (blue), separated airway (cyan), airway NPs (yellow), and acinar NPs (magenta) in a whole mouse

lung enabled through LungVis 1.0. It depicts the lung shown in Fig. 2n and Supplementary Fig. 5b.

Supplementary Movie 6: 3D mapping of a 0h ITLI lung.

Co-visualization of NP distribution (magenta), lung geometry (green) and manually corrected AI airway segments (blue), separated airway (cyan), airway NPs (yellow), and acinar NPs (magenta) in a mouse left lobe (Fig. 3b).

Supplementary Movie 7: 3D mapping of a 2h ITLI lung.

Co-visualization of NP redistribution (magenta), lung geometry (green), and AI-derived airway segments (blue) in a mouse left lobe (Fig. 3f).

Supplementary Movie 8: 3D mapping of a 24h ITLI lung.

Co-visualization of NP redistribution (magenta), lung geometry (green) and AI-derived airway segments (blue) in a mouse left lobe (Fig. 3j).

Supplementary Movie 9: 3D mapping of a 14d ITLI lung.

Co-visualization of NP redistribution (magenta), lung geometry (green) and AI-derived airway segments (blue) in a mouse left lobe (Fig. 3n).

Supplementary Movie 10: 3D mapping of a 0h VAAD lung.

Co-visualization of NP distribution (magenta), lung geometry (green) and manually corrected AI airway segments (blue), separated airway (cyan), airway NPs (yellow), and acinar NPs (magenta) in a mouse left lobe (Fig. 4b).

Supplementary Movie 11: 3D mapping of a 2h VAAD lung.

Co-visualization of NP redistribution (magenta), lung geometry (green) and manually corrected AI airway segments (blue) in a mouse left lobe (Fig. 4f).

Supplementary Movie 12: 3D mapping of a 24h VAAD lung.

Co-visualization of NP redistribution (magenta), lung geometry (green) and AI-derived airway segments (blue) in a mouse left lobe (Fig. 4j).

Supplementary Movie 13: 3D mapping of a 14d VAAD lung.

Co-visualization of NP redistribution (magenta), lung geometry (green), and AI-derived airway segments (blue) in a mouse left lobe (Fig. 4n).

Supplementary Movie 14: Multiple localizations of macrophages in a 2h ITLI lung.

Cellular-resolution visualization of multiple locations of anti-F4/80^{+}, NP-laden macrophages

particularly the ones located in interalveolar pores in 3D immunostained PCLS post 2h ITLI (Fig. 5g and Supplementary Fig. 12a). DAPI, anti-F4/80, podoplanin, and NPs as well as their corresponding surface renderings are in blue, green, cyan (transparent gray in surface rendering), and magenta, respectively.

Supplementary Movie 15: Multiple localizations of macrophages in a 2h VAAD lung.

Cellular-resolution visualization of multiple locations of anti-F4/80^{+}, NP-laden macrophages particularly the ones located in interalveolar pores in 3D immunostained PCLS post 2h VAAD (Fig. 5h and Supplementary Fig. 12d). DAPI, anti-F4/80, podoplanin, and NPs as well as their corresponding surface renderings are in blue, green, cyan (transparent gray in surface rendering), and magenta, respectively.

Supplementary Movie 16: Lung intravital microscopy of a 0h VAAD lung.

Lung intravital microscopy depicts the crawling dynamics of PKH^{+}NP^{+} and PKH^{+}NP^{-} macrophages in living mice for 2h immediately after VAAD delivery (PKH^{+} cells in green, NPs in magenta, Fig. 6a).

Supplementary Movie 17: *Ex-vivo* 4D living tissue microscopy of a 0h ITLI lung.

Ex-vivo 4D living tissue microscopy depicts the crawling behaviors of CSF1R-EGFP^{+}NP^{+} and CSF1R-EGFP^{+}NP^{-} macrophages in living lung sections (Fig. 6f). Macrophages in green and NPs in magenta.

Supplementary Movie 18: Co-mapping of NP distribution with lung phagocytes.

Cellular resolution visualization of CSF1R-EGFP^{+}NP^{+} and CSF1R-EGFP^{+}NP^{-} macrophages in a whole-mount immunostained intact lung lobe (Fig. 6j). Macrophages in green and NPs in magenta.

Supplementary Movie 19: Co-mapping of NP distribution with lung vasculature network.

Cellular resolution visualization of the vascular system (LYVE1^{+}) and NP distribution in a whole mount immunostained intact lung lobe. LYVE1^{+} vessels in cyan and NPs in red (Fig. 7e).
