# Supplementary material for: LungVis 1.0: an automatic AI-powered 3D imaging ecosystem unveils spatial profiling of nanoparticle delivery and acinar migration of lung macrophages
Source: Nat Commun. 2024 Nov 27;15:10138. doi: 10.1038/s41467-024-54267-1 (PMC11603200; doi:10.1038/s41467-024-54267-1)
Supplement: Supplementary file 3 — Reporting Summary [file 41467_2024_54267_MOESM3_ESM.pdf]

nature portfolio

Corresponding author(s): Lin Yang, Otmar Schmid

Last updated by author(s): Oct 7, 2024

Reporting Summary

Nature Portfolio wishes to improve the reproducibility of the work that we publish. This form provides structure for consistency and transparency in reporting. For further information on Nature Portfolio policies, see our Editorial Policies and the Editorial Policy Checklist.

Statistics

For all statistical analyses, confirm that the following items are present in the figure legend, table legend, main text, or Methods section.

n/a	Confirmed	
☐	☒	The exact sample size (*n*) for each experimental group/condition, given as a discrete number and unit of measurement
☐	☒	A statement on whether measurements were taken from distinct samples or whether the same sample was measured repeatedly
☐	☒	The statistical test(s) used AND whether they are one- or two-sided<br>*Only common tests should be described solely by name; describe more complex techniques in the Methods section.*
☐	☒	A description of all covariates tested
☐	☒	A description of any assumptions or corrections, such as tests of normality and adjustment for multiple comparisons
☐	☒	A full description of the statistical parameters including central tendency (e.g. means) or other basic estimates (e.g. regression coefficient) AND variation (e.g. standard deviation) or associated estimates of uncertainty (e.g. confidence intervals)
☐	☒	For null hypothesis testing, the test statistic (e.g. *F*, *t*, *r*) with confidence intervals, effect sizes, degrees of freedom and *P* value noted<br>*Give P values as exact values whenever suitable.*
☒	☐	For Bayesian analysis, information on the choice of priors and Markov chain Monte Carlo settings
☐	☒	For hierarchical and complex designs, identification of the appropriate level for tests and full reporting of outcomes
☐	☒	Estimates of effect sizes (e.g. Cohen's *d*, Pearson's *r*), indicating how they were calculated

Our web collection on statistics for biologists contains articles on many of the points above.

Software and code

Policy information about availability of computer code

Data collection	1. All light sheet fluorescence microscopy data for tissue-cleared murine lungs was collected through Ultramicroscope II, LaVision Biotec equipped with a sCMOS camera (Andor Neo) and a 2× objective lens (Olympus MVPLAPO 2×/0.5 NA) equipped with an Olympus MVX-10 zoom body.<br>2. Confocal imaging of precision cut lung slices (PCLS) and ex vivo living tissue was performed on an LSM710 system (Zeiss) containing an inverted AxioObserver.Z1 stand equipped with phase-contrast and epi-illumination optics and operated by ZEN2009 software (Zeiss). Ex vivo living lung tissue imaging was also performed on light-sheet fluorescence microscope (Lightsheet Z.1 equipped with an incubation system, Zeiss).<br>3. Lung intravital microscopy was performed with a VisiScope.A1 imaging system (Visitron Systems GmbH, Puchheim, Germany), equipped with an LED light source for fluorescence epi-illumination (pe-4000, CoolLed, Andover, UK).<br>4. Flow cytometry was determined using BD FACSFortessa running FACSDiva software v8.0.1. Cell types differentiation and particle quantifications were performed using FlowJo v10.8.1.<br>5. PCLS was cut with a vibratome (Hyrax V55, Zeiss, Germany) to a thickness of 300 µm.<br>6.Lung homogenates was obtained using a disperser (T10 basic ULTRA-TURRAX®) and Nanoparticle fluorescence intensity was determined with a standard multi-well plate reader (Tecan Safire 2) at the optical. wavelength of 635/685 nm with a 10 nm bandwidth of optical filters<br>7. Ventilator-assisted aerosol delivery (VAAD) was performed with a mechanical ventilator (flexiVent FX system, Scireq Inc., Canada) equipped with a nebulizer (Aeroneb Lab Small, Aerogen Inc., Galway, Ireland).<br>8. Nose-only aerosol inhalation (NOAI) was performed with a a Pari Sprint LC nebulizer delivered aerosols to mouse nostrils via an in-house newly developed nose-only inhalation system.
Data analysis	Flow cytometry data were analyzed with FlowJo v10.8.1.<br>Numerical data analysis, visualization, and statistical tests were performed with in Microsoft Excel, GraphPad Prism v9, Python 3.8.

Our AI code for LSFM imaging data is publicly available (stated below in the Data session). The following open source libraries have been utilized as part of our pipelines (Python 3.8) packages to be installed via "pip install <package name>"): Pytorch=1.11.0, git+https://github.com/MIC-DKFZ/batchgenerators#6859efd8cd59000896c0bcb6313e2b8e12bbb031
Moreover, we utilized and modified nnU-Net, detailed installation instructions can be found in our code repository.
Schematic illuminations were drawn in Biorender.
Detailed data analysis and statistical tests were documented in the Materials and methods sections as well as in Figure legends.

For manuscripts utilizing custom algorithms or software that are central to the research but not yet described in published literature, software must be made available to editors and reviewers. We strongly encourage code deposition in a community repository (e.g. GitHub). See the Nature Portfolio guidelines for submitting code & software for further information.

Data

Policy information about availability of data

All manuscripts must include a data availability statement. This statement should provide the following information, where applicable:

- Accession codes, unique identifiers, or web links for publicly available datasets
- A description of any restrictions on data availability
- For clinical datasets or third party data, please ensure that the statement adheres to our policy

The raw LSFM lung images generated from LSFM, reference lung annotations, and AI-generated airway models obtained from LungVis 1.0 are publicly available via Zenodo (https://doi.org/10.5281/zenodo.7413818). Computing codes for the data-centric active learning AI training model are provided via GitHub (https://github.com/MIC-DKFZ/MurineAirwaySegmentation). Any remaining data supporting the findings from this study are available from the corresponding authors upon reasonable request.

Research involving human participants, their data, or biological material

Policy information about studies with human participants or human data. See also policy information about sex, gender (identity/presentation), and sexual orientation and race, ethnicity and racism.

Reporting on sex and gender	This study does not involve human research participants
Reporting on race, ethnicity, or other socially relevant groupings	N/A.
Population characteristics	N/A.
Recruitment	N/A.
Ethics oversight	N/A.

Note that full information on the approval of the study protocol must also be provided in the manuscript.

Field-specific reporting

Please select the one below that is the best fit for your research. If you are not sure, read the appropriate sections before making your selection.

☒ Life sciences ☐ Behavioural & social sciences ☐ Ecological, evolutionary & environmental sciences

For a reference copy of the document with all sections, see nature.com/documents/nr-reporting-summary-flat.pdf

Life sciences study design

All studies must disclose on these points even when the disclosure is negative.

Sample size	No statistical method was used to predetermine sample size.<br>Time-resolved different routes of pulmonary delivery included 3-8 mice per group. All other animal relevant experiments including intravital microscopy, ex live living tissue microscopy, flow cytometry, immunofluorescence staining were done with at least 3 mice/lungs per group.<br>Sample size was considered sufficient based on the high reproducibility of the results.
Data exclusions	No data were excluded from the manuscript.
Replication	All results reported in this paper were consistently reproduced using a minimum of 3 mice. The sample size and number of replicates for each experiment are detailed in the figure legends. Detailed information on each experiment can be found in the Methods section. Additionally, the raw Light Sheet Fluorescence Microscopy (LSFM) imaging data and the AI-generated models of lung airways from more than 80 mouse lungs have been made publicly available.
Randomization	For all experiments, mice were randomly split into groups with different routes of pulmonary delivery and various time points of treatment.

Blinding	Blinding was not relevant to this study since the experimental groups have to be randomly assigned at the beginning of the experiment and the treatment conditions are also evident from the imaging data.

Reporting for specific materials, systems and methods

We require information from authors about some types of materials, experimental systems and methods used in many studies. Here, indicate whether each material, system or method listed is relevant to your study. If you are not sure if a list item applies to your research, read the appropriate section before selecting a response.

Materials & experimental systems

n/a	Involved in the study
☐	☒ Antibodies
☐	☒ Eukaryotic cell lines
☒	☐ Palaeontology and archaeology
☐	☒ Animals and other organisms
☒	☐ Clinical data
☒	☐ Dual use research of concern
☒	☐ Plants

Methods

n/a	Involved in the study
☒	☐ ChIP-seq
☐	☒ Flow cytometry
☒	☐ MRI-based neuroimaging

Antibodies

Antibodies used

Immunofluorecence staining
anti-F4/80 (ab90247, 1:100, rat; Abcam)
Cd11b (ab133357, 1:2000, rabbit, Abcam)
Cd11c (#97585, 1:200, rabbit, Cell signaling)
Cd68 (ab125212, 1:100, rabbit, Abcam)
Cd45 (ab10558, 1:250, rabbit, Abcam)
LYVE-1 (ab14917, 1:100, rat; Abcam)
Podoplanin (AF3244, 1:200, goat; R&D systems)
a-SMA (A5228, 1:5,000, mouse; Sigma)
Anti-GFP (ab13970, 1:1000, chicken, Abcam)
Alexa Fluor™ 594 Phalloidin (A12381, 1:300; Invitrogen).
Goat anti-mouse IgG Alexa Fluor-488 (1:200; A-11001, Invitrogen)
Goat anti-rat IgG Alexa Fluor-488 (1:200; A-11006, Invitrogen)
Donkey anti-rabbit Alexa Fluor 488, (1:200, A21206, Invitrogen)
Donkey anti-mouse IgG Alexa Fluor-488 (1:200; A-21202, Invitrogen)
Donkey anti-rat IgG Alexa Fluor-488 (1:200; A-21208, Invitrogen)
Donkey anti-goat IgG Alexa Fluor-568 (1:200; A-11057, Invitrogen)
Goat anti-chicken IgG Alexa Fluor-488 (1:200; A-11039, Invitrogen)

Flow cytometry
purified anti-mouse CD16/CD32, clone 93, 1:100 Cat. No. 14-0161-82, ThermoFisher
PE-conjugated anti-CD11b, clone: REA592, 1: 50, Cat. No. 130-113-806, Miltenyi Biotec
FITC-CD11c, clone: REA754, 1: 50, Cat. No. 130-110-837, Miltenyi Biotec
PE-Vio® 770-conjugated Siglec-F, clone: REA798, 1: 50, Cat. No. 130-112-334, Miltenyi Biotec
PE-Vio® 770-conjugated CD11b, clone: REA592, 1: 50, Cat. No. 130-113-808, Miltenyi Biotec

Validation

All antibodies used in this study were purchased from commercial vendors who had validated specificity in mouse tissues/cells for the specific assays (immunofluorescence and flow cytometry). Detail information of commercial antibodies can be checked from corresponding websites. We have validated the efficiency of each antibody by setting up multiple negative controls. For example, to validate the specificity of IF antibodies, we employed non-stained, only primary Ab stained, or only secondary Ab stained samples as controls.

Eukaryotic cell lines

Policy information about cell lines and Sex and Gender in Research

Cell line source(s)	MH-S cell line was obtained from ATCC.
Authentication	none of cell lines used were authenticated
Mycoplasma contamination	The cell line tested negative for Mycoplasam contamination
Commonly misidentified lines (See ICLAC register)	No commonly misidentified cell lines were used

Animals and other research organisms

Policy information about studies involving animals; ARRIVE guidelines recommended for reporting animal research, and Sex and Gender in Research

Laboratory animals	Wildtype (WT) C57BL/6 female and male mice were purchased form Charles River, Germany and were bred in house. WT mice with age 9 - 18 weeks and 19 - 25 g of body weight (BW) were housed in individually ventilated cages (IVC-Racks; Bio-Zone, Margate, UK) supplied with filtered air in a 12h light / 12h dark cycle. Mice were provided with food (standard chow) and water ad libitum. The MacGreen colony (CSF1R-EGFP, strain # 018549, age 10–12 weeks) was maintained as homozygote and all offspring were positive for an enhanced green fluorescent protein that was subsequently used for ex vivo living tissue imaging and tissue-cleared LSFM.
Wild animals	No wild animals were used.
Reporting on sex	Both male and female mice were used.
Field-collected samples	The study did not involve samples collected from the field.
Ethics oversight	All procedures involving animal handling and experiments were carried out in accordance with protocols approved by the Regierung von Oberbayern, Germany (District Government of Upper Bavaria, AZ55.2-1-54-2532-108.13 and AZ55.2-1-54-2532-67-2015) and were performed according to federal and institutional guidelines.

Note that full information on the approval of the study protocol must also be provided in the manuscript.

Plants

Seed stocks	*Report on the source of all seed stocks or other plant material used. If applicable, state the seed stock centre and catalogue number. If plant specimens were collected from the field, describe the collection location, date and sampling procedures.*
Novel plant genotypes	*Describe the methods by which all novel plant genotypes were produced. This includes those generated by transgenic approaches, gene editing, chemical/radiation-based mutagenesis and hybridization. For transgenic lines, describe the transformation method, the number of independent lines analyzed and the generation upon which experiments were performed. For gene-edited lines, describe the editor used, the endogenous sequence targeted for editing, the targeting guide RNA sequence (if applicable) and how the editor was applied.*
Authentication	*Describe any authentication procedures for each seed stock used or novel genotype generated. Describe any experiments used to assess the effect of a mutation and, where applicable, how potential secondary effects (e.g. second site T-DNA insertions, mosiacism, off-target gene editing) were examined.*

Flow Cytometry

Plots

Confirm that:

☒ The axis labels state the marker and fluorochrome used (e.g. CD4-FITC).

☒ The axis scales are clearly visible. Include numbers along axes only for bottom left plot of group (a 'group' is an analysis of identical markers).

☒ All plots are contour plots with outliers or pseudocolor plots.

☒ A numerical value for number of cells or percentage (with statistics) is provided.

Methodology

Sample preparation	Lungs were transferred for enzymatic digestion for 30 min at 37°C in a mixture of dispase (50 caseinolytic U/mL), collagenase (2 mg/mL), elastase (1 mg/mL), and DNase (30 µg/mL). Single cell suspensions were harvested by softly smashing tissue through 100 µm mesh using the plunger of a 3 mL syringe and then followed with filtering through a 40-µm mesh. After centrifugation at 300g for 5 minutes, red blood cells were removed with 1mL lysis buffer for 30-40s. Single lung cells were then generated with addition of 5-6 mL RPMI+10%FCS to the sample tube, washed in 1 ml 1x fluorescence-activated cell sorting (FACS) buffer, and centrifuged for 5 mins at 300g at 4°C, and counted for total cell numbers and overall cell viability as described above. The single cell numbers were counted at most 1 million per 100 µL FACS buffer/tube and incubated with 50 µL Fc block (CD16/CD32, 1:100 in FACS buffer) for 20 mins at 4°C. After 30 min incubation with antibody suspension for 30 mins at 4°C, the single cell suspensions in 200 µL of MACS were then analyzed using BD FACSFortessa running FACSDiva software v8.0.1.
Instrument	BD FACSFortessa running FACSDiva software v8.0.1
Software	FlowJo v10.8.1 was used to analyze the cell populations and NP quantifications
Cell population abundance	After sorting out the deris, the living cells were grated for NP+ cells (0.89%-1.27%), PKH+ cells (1.01%-2.01%), and GFP+ cells (18%-28%).

Gating strategy

Grating strategies have been included in multiple figures (Figure 5i, Figure 6g, supplementary Figure 13d, and supplementary Figure 14b). Deris were excluded by FSC-A vs SSC-A. For further gating of specific cell populations, boundaries were selected based on the negative and positive staining.

☒ Tick this box to confirm that a figure exemplifying the gating strategy is provided in the Supplementary Information.
